# Parents’ Participation in Care during Neonatal Intensive Care Unit Stay in COVID-19 Era: An Observational Study

**DOI:** 10.3390/nursrep14020092

**Published:** 2024-05-13

**Authors:** Emanuele Buccione, Davide Scarponcini Fornaro, Damiana Pieragostino, Luca Natale, Adelaide D’Errico, Valentina Chiavaroli, Laura Rasero, Stefano Bambi, Carlo Della Pelle, Susanna Di Valerio

**Affiliations:** 1 Department of Biomedicine and Prevention, University of Rome Tor Vergata, 00133 Rome, Italy; 2 Neonatal Intensive Care Unit, Health Local Authority 3 Pescara, 65124 Pescara, Italy; davide.scarponcini@asl.pe.it (D.S.F.); valentina.chiavaroli@asl.pe.it (V.C.); susanna.divalerio@asl.pe.it (S.D.V.); 3 Department of Innovative Technologies and Medicine & Odontoiatry, University G. D’Annunzio, Chieti-Pescara, 66100 Chieti, Italy; damiana.pieragostino@unich.it (D.P.); luca.natale@unich.it (L.N.); 4 Analytical Biochemistry and Proteomics Laboratory, Center for Advanced Studies and Technology (CAST), “G. d’Annunzio” University of Chieti-Pescara, 66100 Chieti, Italy; 5 Neonatal Intensive Care Unit, Santobono-Pausilipon Children’s Hospital, 80129 Naples, Italy; a.derrico@santobonopausilipon.it; 6 Department of Health Sciences, University of Florence, 50134 Florence, Italy; l.rasero@unifi.it (L.R.); stefano.bambi@unifi.it (S.B.); 7 Medical Department, Health Local Authority 2 Chieti, 66100 Chieti, Italy; carlo.dellapelle@unich.it

**Keywords:** neonatal intensive care unit, parenthood, newborn, preterm, nurse

## Abstract

Background: Parents play a crucial role in the care of infants during their stay in the neonatal intensive care unit (NICU). Recent studies have reported a decrease in parental participation due to the coronavirus disease (COVID-19) pandemic, which has led to restricted access policies in hospitals. The aim of this study was to describe the barriers to good parental participation during their stay in the neonatal intensive care unit in the COVID-19 era. Methods: This was a quantitative, observational study. Results: A total of 270 parents participated in this study. Mothers’ participation in care was higher than that of fathers (*p* = 0.017). Parents who lived at the birth of their first child reported a better level of participation in care compared to those who lived at the birth of their second-born (*p* = 0.005). Parents of extremely preterm neonates reported a lower interaction with their infants than parents of term newborns (*p* < 0.001). Conclusions: Some disadvantaged categories reported lower scores for cultural and linguistic minorities, parents of multiple children, and fathers. The COVID-19 pandemic has made several family-centred care activities impossible, with a higher impact on those who benefited most of these facilities. This study was prospectively approved by the IRB-CRRM of the University “G. d’Annunzio” Chieti-Pescara on 23 January 2024 (approval number CRRM: 2023_12_07_01).

## 1. Introduction

The length of stay of preterm and ill newborns in the neonatal intensive care unit (NICU) can be considerably extended [1]. This period often causes separation between parents and their offspring, limiting emotional and physical closeness [2]. Furthermore, the period after birth is critical for bonding between parents and newborns [3]. Parents’ participation in care and their relationship with their babies are fundamental to infant health and neurobehavioral development [4]. During their stay in the NICU, parents play a crucial role in the care of their infants [5]. Models such as family-centred care (FCC) and family integrated care (FICare) promote parental participation [6]. These programmes allow parents to become confident, knowledgeable, and independent primary caregivers. FCC and FICare can shorten the time needed to use the nasogastric tube, reduce the length of stay in hospital, increase the rate of exclusive breastfeeding, improve the overall prognosis of preterm infants, and exert positive effects on parents [7].

Furthermore, previous studies have shown that alterations in their role are the greatest source of stress for parents [8,9,10]. A recent meta-analysis showed that parental stress related to NICU admission is a worldwide healthcare issue. Immediate support for parents should be prioritized to reduce parental stress and promote the emotional well-being of mothers and fathers [11].

Before the COVID-19 pandemic, the integration of parents into their infants’ care began immediately upon admission. Parents were empowered to provide as much of their infants’ care as possible. Parents participated in the educational sessions and were supported by nursing staff to actively participate in the daily decision-making process [12]. Unfortunately, recent studies have reported a decrease in parental presence and participation during the NICU stay due to the coronavirus disease (COVID-19) pandemic, which determined restricted access policies in hospitals [13,14,15]. Restricted visit policies have resulted in a negative experience of parenthood, emotional struggles, feelings of isolation, lack of family-centred care, deep disappointment with system-level decisions, and a negative impact on breastfeeding [14,16]. When parents in the NICU are distressed or depressed, their interactions with their infants may be less sensitive and attuned to their infants’ needs [17]. For this reason, additional attention and support were necessary for parents in the NICU during the COVID-19 pandemic, given its association with increased stress and a potential impact on family outcomes [18].

Health practitioners, healthcare organizations, and health systems need to be engaged in working towards cultural safety and critical consciousness [19]. It was also described that the COVID-19 pandemic has had a disproportionate impact on vulnerable populations. Any ethnic and cultural disparities in the NICU must be addressed to allow all families equal opportunities for collaboration, decision making, planning, information sharing, and participation in the care of their children, whether it occurs in person or remotely [20].

The impact of COVID-19 restrictions on parents’ access to NICU has been qualitatively described in several published studies [13,14,15], but none have quantitatively reported the limits related to decreased participation in care. In the Italian healthcare context, no instruments are available to quantitatively evaluate participation in NICU activities. Recently, Scarponcini Fornaro et al. validated the Italian version of the scale ‘Parental Participation in Care: Neonatal Intensive Care Unit (PPCS: NICU)’, which allows healthcare professionals to evaluate parents’ participation in the care of their neonates [21,22]. The aim of this study was to describe the barriers to good parental participation during their stay in the NICU in the COVID-19 era.

## 2. Materials and Methods

A monocentric retrospective cohort study was designed following the statement ‘Strengthening the Reporting of Observational Studies in Epidemiology (STROBE)’ [23].

### 2.1. Sample and Setting

The study was conducted in 22-bed mixed NICU (medical and surgical) of a generic hospital. The study participants included parents whose babies were admitted to the NICU and who agreed to participate using a written informed consent form. Parents who disagreed with participation or those younger than 18 years of age were excluded. During data collection, one parent at a time was allowed to enter the NICU twice a day, for a maximum of two hours, owing to visiting restrictions related to COVID-19. For twins, both parents were allowed to enter the NICU simultaneously. During the pre-pandemic period, the NICU was opened 24 h a day for visits from both parents.

### 2.2. Data Collection

The data collected refer to the period between April and December 2022. Parents’ sociodemographic data were collected (age, sex, ethnicity, occupational status, experience of previous abortion, or deaths). Newborns were recorded for gestational age, body weight at admission, type of childbirth, twin birth, and all medical devices used to support the newborn. During the COVID-19 pandemic, NICU nurses took part in an educational course based on a tool with training in its use. Afterwards, they performed evaluations of parental participation in care to highlight parents with low participation. Each parent underwent two evaluations. The first observation was recorded during the first three days. The second observation was performed between the seventh and tenth days of hospitalization. The dataset was stored in the ZENODO consultable using the identifier 10.5281/zenodo.11071027. The instrument used was the Italian PPCSNICU [22]. It allows an evaluation of parental participation in care by Italian nurses or other healthcare professionals independently from the ethnicity of parents cared for and consists of one dimension composed of 16 items, which is similar to the original scale [21]. The items used a 3-point Likert scale (3 = always, 2 = sometimes, and 1 = never). The highest possible score was 48, and the lowest was 16. A score of 16 points indicated that the parent did not participate in caring for her or his infant. Higher scores indicated higher participation levels. No cutoff points were specified by the instrument [21]. The first three items focused on communication between the parents and health professionals. From the fourth to the fifteenth items, the tool covers the interaction between parent and newborn (physical contact, breastfeeding, hygienic care, and support during painful procedures). The last item focused on parents’ expressions of emotions and fears. In a validation study, the Italian tool showed an overall content validity index (CVI) of 0.976 and good reliability (Cronbach’s α = 0.926) [22] (Supplementary Material).

### 2.3. Statistical Analysis

Excel 2021 v16.0 software was used to store the data. Summary statistics are presented as absolute frequencies and percentages and as medians and interquartile ranges [IQRs] for continuous non-normally distributed data (according to the Shapiro–Wilk test). Nonparametric tests were performed to compare the median values reported by each categorical variable using the Kruskal–Wallis test and Mann–Whitney U test. Bonferroni-adjusted *p* values were calculated for multiple comparisons. Subgroup analysis by period was performed to assess whether parental participation differed according to the duration of neonatal hospitalization. We used multivariate linear regression to identify the independent variables associated with changes in parental participation in care. Regression parameters are presented as reporting unstandardized and standardized coefficients and their 95% confidence intervals. The coefficient of determination is described for the model. Statistical significance was established at *p* value less than 0.05. Statistical analyses were performed using the IBM SPSS version 22.0. For statistical power analysis, we used G * Power 3.1 [24].

## 3. Results

Two hundred and seventy parents were included in the study. Of the sample, 50.4% were female (N = 136). A total of 21.9% (n = 59) had previous abortions, while 2.6% (n = 7) had previous offspring deaths. It was the birth of their first child for 55.2% (n = 149), and most couples (47.4%, n = 128) experienced natural childbirth. Most participants were Caucasian (88.1%, n = 238). The median [IQR] patient age was 34 [9] years. The features of all parents are detailed in Table 1.

In this study were included one hundred and fifty-two newborns, of which 11.2% (n = 17) were twins. Of these, 37.1% (n = 50) were preterm infants. The median body weight at admission [IQR] was 2790 [1140] g. The median gestational age was 37 [5] weeks.

The median score for overall participation in care at admission was 41 [10]. After approximately seven days of hospitalization, a significant overall improvement was observed, with a median score of 46 [8] and *p* < 0.001. 

### 3.1. Barriers Related to Parental Background

Mothers’ participation in care levels appeared to be significantly higher than that of fathers (median scores of 42 [8] and 39 [11], respectively; *p* = 0.017). This result was also confirmed by the second observation, when mothers reported a median score of 46 [6] versus 44 [9] reported by fathers (*p* = 0.003). Parents who lived the birth of their first child reported a better median level of participation in care than those who lived the birth of their second child (42 [8] vs. 38 [12]); *p* = 0.005). Furthermore, this difference appeared to remain unchanged over time, with a higher score in parents of only children (46 [6] vs. 44 [10]); *p* = 0.015). African parents reported significantly lower participation in care, with a median score of 30.5 [15] vs. 42 [8] reported by Caucasian people and 45 [8] by Hispanics (*p* < 0.001); this difference remained significant in the second observation. Lastly, parents who experienced natural birth showed higher participation in care with a median score of 43 [8] than parents who underwent emergency cesarean section (median score 38 [12]) (*p* < 0.001); however, the difference was still significant over time.

There was no correlation between age and parenthood (r = −0.80; *p* = 0.18 at admission and r = −0.87; *p*= 0.15 after approximately 7 days). Other factors, such as previous abortions, previous deaths, and occupational status, did not affect the level of parenthood during the stay in the NICU. All scores are presented in Table 1.

### 3.2. Barriers Related to Neonates’ Features

Parents of extremely premature newborns reported a significantly lower interaction with their infants, with a median score of 31 [9], compared to parents of term newborns (median score 42 [9]; *p* < 0.001). However, after approximately seven days of hospitalization, there was no significant difference between the parental participation scores of preterm and term neonates. Parents of twins showed significantly higher participation only in the second evaluation, with a median value of 45 [8] vs. 47 [1] reported by parents of only newborns (*p* = 0.029). According to the Mann–Whitney U test, fathers reported a higher participation than those of only neonates in care, with a median value of 44 [8] vs. a median value of 42 [13] (*p* = 0.025).

Lastly, all devices related to critically ill conditions were associated with significantly lower interactions between parents and neonates. The only devices not associated with a minor interaction were the peripheral venous catheter, high-flow nasal cannula, and monitoring of brain function (Table 2).

### 3.3. Multiple Linear Regression

Multiple linear regression analyses were performed to highlight the association between the independent variables and parental participation in care (Table 3).

It was confirmed that participation in care is negatively associated with the male gender, the need or choice of cesarean section, African ethnicity, and experience of previous abortions. Furthermore, some clinical devices or conditions could negatively affect the interaction between parents and their newborns: a very low weight, a high degree of prematurity, a need for invasive ventilation, the placement of a bladder catheter, or the presence of stoma.

### 3.4. Sensitivity Power Analysis of Multiple Linear Regression

**Figure 1:**
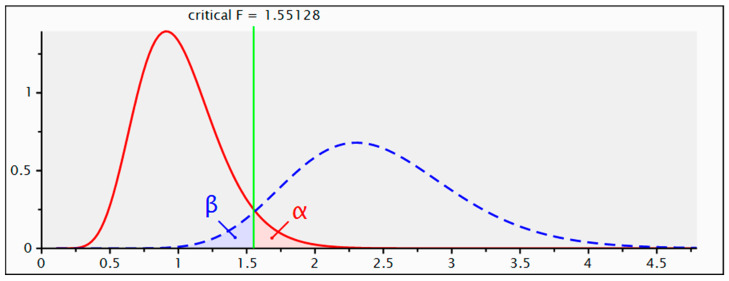
Distribution plot.

Sensitivity power analysis allowed for the determination of the minimum effect size to which this study was sensitive. For a power of 0.95, based on the recruited sample size (270 participants) and an alpha level of 0.05, this analysis showed sensitivity for an effect size f2 = 0.13, which is defined as medium (Figure 1).

## 4. Discussion

This study described the early barriers to parental participation in care during their stay in NICU during the COVID-19 pandemic. No similarly published study had a comparable sample size which has been shown to be sensitive to a medium effect size [25]. Furthermore, this is the first study to use the PPS:NICU scale after a cross-cultural validation of the Italian population. This scale allows for the quantitative assessment of the participation of nurses and health professionals. Previously, only the Italian EMpowerment of PArents in the Intensive Care-Neonatology (EMPATHIC-N) questionnaire was available, but this tool provides a self-assessment of the quality of care perceived by parents of neonates admitted to the NICU [26]. Another study used the index parental participation scale (IPP), but no validation study was carried out to test the Italian version [27].

The sample highlighted fair general participation in care, according to another Italian study that reported no significant differences in participation before and during the pandemic [27]. Despite this, our results highlight an important cultural barrier to participation in care, as shown by foreign parents, as described in previous studies. In fact, minority families face multiple barriers to engaging in collaborative partnerships with providers: systemic racism, stereotyping, and inadequate attention to the social determinants of health [28]. Furthermore, a low awareness of cultural and social factors by healthcare professionals can reduce the effectiveness of communication with families in the NICU, exacerbate family denial, erode trust, and generally have a damaging effect on interactions between staff and families [29,30]. Lastly, the impact of parental primary language on communication in the neonatal intensive care unit was also described as a factor contributing to suboptimal healthcare delivery [31]. It is likely that the limitations on visits caused by the policy restrictions due to COVID-19 and conversations between parents and healthcare professionals often conducted by phone made the impact of these barriers very large. This result is supported by a qualitative observational study that focused on parents’ and neonatal healthcare professionals’ views on barriers to parental presence in the NICU. Schimd et al. described that parental presence was influenced by communication, relationship, and interaction in infant care, as well as cultural aspects and language [32].

Parents who have already had other children show a lower participation score, which could be due to the lack of baby-sitting, as already described by Kerr et al., and can be linked to a poor availability of babysitting services for other children [33]. Furthermore, during the pandemic period, there was the fear of attending the NICU because they risked having contracted the virus and infecting the closest relatives.

Our findings also highlight a lower interaction between extremely preterm newborns and their parents. A previously published study described that, during the stay in the NICU, mothers of preterm infants experienced the disruption of family dynamics, support and bonding, physical and emotional isolation, a negative psychological impact compounded by increased concerns, a change in maternal role, and survival mode mentality [34]. A meta-analysis including 38 studies and a total of 3025 parents of preterm infants indicated that parents of preterm-born children experienced only slightly more stress than parents of term-born children. Parents report more stress in infants with lower gestational age and lower birth weights [35]. Furthermore, the complex psychosocial needs of parents of extremely preterm infants were challenging for the NICU and its staff before the COVID-19 pandemic [36]. Communicating parents’ needs and informing them about the available support is essential in order to help them cope with their infants’ hospitalization [36]. However, restrictions during the COVID-19 era have often made this impossible. It is already described that, during the pandemic period, parents experienced increased stress due to the restricted NICU visitation policies, limited opportunities to care for their infant, lack of support, and inconsistent communication regarding their infant status and COVID-19 protocols [37].

Medical equipment, such as devices for respiratory support, bladder catheter, and arterial access, may constitute barriers to the parents in their wish to see and touch their child. Indeed, our results are supported by a previously published survey that focused on parental interactions with infants treated with medical devices. The authors described that all types of medical technology were perceived as significantly obstructive in all aspects, except for feeding tubes. Furthermore, Lantz and Ottosson found that monitoring, phototherapy, and continuous infusion (which are some of the same barrier factors described by our study) were perceived by parents as significantly greater obstacles to their wish to touch their child, compared with their wish to see their baby [38].

According to our results, fathers seemed less involved than mothers in caring for their newborns. This is supported by another result that shows how the fathers of the twins, who had the opportunity to visit their children together with their partner, reported a higher value of participation than the fathers of only children. As previously described, this is reportable to relational suffering (separation from the partner and newborn) [39]. Furthermore, fathers who experienced minor restrictions reported greater involvement in caregiving activities [40]. A previous qualitative study described three themes: the need for support, clarity, and recognition. Fathers have specific needs that must be addressed. An awareness of addressing fathers’ needs promotes more holistic care, supports coping within the NICU environment, and helps fathers engage in the care of their infant [41]. Furthermore, a recent study highlighted how early positive perceptions of fatherhood could significantly predict fathers’ confidence in neonatal care and be significantly influenced by psychological satisfaction owing to the intimate relationship between fathers and their offspring [42].

Lastly, this study showed a lower involvement of parents who had planned or unplanned cesarean sections. Two previously published studies have supported these findings. Mothers who experienced cesarean section reported worse postnatal depression, lower maternal bonding, and less openness [43]. Furthermore, cesarean sections cause maternal feelings such as sadness and disappointment with the unplanned birth process [44].

Before the pandemic, implementing parent–infant closeness in the NICU was a challenge for nurses and healthcare professionals [1]. Optimization in neonatal care, such as zero separation and parent–infant closeness, was reset with the onset of the pandemic. The ideal collaboration between NICU nurses and parents has always been characterized by flexibility and reciprocity and is based on verbal and action dialogues [5]. Obviously, during the pandemic, this was very limited, with a negative impact on the well-being of parents and newborns.

This study had several limitations. Owing to convenience sampling, the sample may not be representative of the general population and is prone to selection bias. The monocentric nature of our study affected the sample size. Finally, the retrospective design is prone to misclassification bias.

## 5. Conclusions

Many studies have provided qualitative analyses of the feelings and emotions experienced by parents of infants admitted to the NICU during the COVID-19 pandemic. This study is one of the few to provide a quantitative description of the interactions between parents and their newborns. This was possible using the Italian PPCS:NICU scale, which was validated in the Italian context. Despite fairly good participation in care, some barriers to parenthood during the NICU stay in the COVID-19 era were highlighted. Some more disadvantaged categories reported lower scores: parents of cultural and linguistic minorities, parents of multiple children, and fathers. The COVID-19 pandemic made several family-centred care activities impossible, with a greater impact on those who benefited the most from these facilities (24 h visit, kangaroo care, cultural mediation service, and psychological or educational support).

## Figures and Tables

**Table 1:** Median scores according to characteristics of parents and newborns.

Factor	Categories	N (%)	Median [IQR] I Assessment	*p*-Value	Median [IQR] II Assessment	*p*-Value
Gestational Age	Extremely Preterm	8 (3.0%)	31 [9]	**<0.001**	39.5 [9]	0.033 †
	Very Preterm	32 (11.9%)	35 [12]		42 [10]	
	Moderate/Late preterm	60 (22.2%)	42.5 [7]		46 [3]	
	Term	170 (63.0%)	42 [9]		45 [7]	
Parents’ Gender	Female	136 (50.4%)	42 [8]	**0.017**	46 [6]	**0.003**
	Male	134 (49.6%)	39 [11]		44 [9]	
First Child	No	121 (44.8%)	38 [12]	**0.005**	44 [10]	**0.015**
	Yes	149 (55.2%)	42 [8]		46 [6]	
Previous Abortions	No	211 (78.1%)	41 [9]	0.325	45 [8]	0.294
	Yes	59 (21.9%)	39 [12]		47 [7]	
Previous Deceased Children	No	263 (97.4%)	41 [10]	0.070	46 [7]	0.093
	Yes	7 (2.6%)	35 [6]		40 [6]	
Type of Delivery	Natural Birth	128 (47.4%)	43 [8]	**<0.001**	46 [6]	**0.006**
	Elective Cesarean Section	76 (28.1%)	40.5 [10]		44 [8]	
	Emergency Cesarean Section	66 (24.4%)	38 [12]		46 [9]	
Parents’ Ethnicity	Caucasian	238 (88.1%)	42 [8]	**<0.001**	46 [7]	**<0.001**
	African	18 (6.7%)	30.5 [15]		38 [9]	
	Hispanic/Latino Americans	10 (3.7%)	45 [8]		47.5 [4]	
	Asian *	4 (1.5%)	16 [0]		16 [0]	
Job	Unemployed	60 (22.2%)	42 [10]	0.119	44 [8]	0.126
	Employee	200 (74.1%)	41 [10]		46 [7]	
	Student	10 (3.7%)	32 [24]		42 [21]	
Twins	No	237 (87.8%)	41 [11]	0.981	45 [8]	**0.029**
	Yes	33 (12.2%)	40 [7]		47 [1]	

Bold values: statistically significant; * = excluded due to low number; IQR: interquartile range; † = not statistically significant after Bonferroni correction.

**Table 2:** Median scores according to the presence of medical device used to support newborns.

Factor	Categories	N (%); Median (IQR) I Assessment	*p*-Value	N (%); Median (IQR) II Assessment	*p*-Value
Peripheric venous catheter	No	119 (44.1%); 39 (11)	0.062	89 (33.0%); 46 (9)	0.663
	Yes	151 (55.9%); 42 (9)		181 (67.0%); 46 (6)	
Central venous catheter	No	238 (88.1%); 41.5 (9)	**0.011**	212 (78.5%); 46 (7)	**<0.001**
	Yes	32 (11.9%); 38 (17)		58 (21.5%); 42 (9)	
Umbilical venous catheter	No	180 (66.7%); 42 (9)	0.462	270 (100%); 46 (8)	-
	Yes	90 (33.3%); 39 (10)		-	
Continuous infusions	No	92 (34.1%); 38.5 (11)	0.203	132 (48.9%); 46 (8)	**0.015**
	Yes	178 (65.9%); 42 (10)		138 (51.1%); 45 (7)	
Arterial catheter	No	260 (96.3%); 41 (10)	**0.032**	270 (100%); 46 (8)	-
	Yes	10 (3.7%); 34 (10)		-	
High-flow nasal cannula	No	226 (83.7%); 41 (11)	0.679	226 (83.7%); 46 (8)	0.713
	Yes	44 (16.3%); 41 (10)		44 (16.3%); 46 (5)	
Non-invasive ventilation	No	258 (95.6%); 41 (10)	0.802	261 (96.7%); 46 (7)	**0.049**
	Yes	12 (4.4%); 41 (7)		9(3.3%); 40 (6)	
Endotracheal tube	No	224 (86.0%); 42 (8)	**0.001**	243 (90.0%); 46 (7)	**0.001**
	Yes	46 (17.0%); 35 (13)		27 (10.0%); 41 (9)	
High-frequency oscillatory ventilation	No	258 (95.9%); 41.5 (9)	**0.005**	266 (98.5%); 46 (8)	0.140
	Yes	12 (4.4%); 34 (10)		4 (1.5%); 40 (10)	
Gastric tube	No	168 (62.2%); 42.5 (10)	**0.006**	170 (63.0%); 46 (7)	0.031
	Yes	102 (37.8%); 39 (11)		100 (37.0%); 44 (8)	
Cerebral function monitoring	No	252 (93.3%); 42 (10)	0.100	264 (97.8%); 46 (8)	0.074
	Yes	18 (6.7%); 39.5 (9)		6 (2.2%); 42 (9)	
Bladder catheter	No	250 (92.6%); 42 (9)	**<0.001**	252 (93.3%); 46 (7)	**<0.001**
	Yes	20 (7.4%); 34 (17)		18 (6.7%); 39 (11)	
Stoma	No	266 (98.5%); 41 (10)	-	264 (97.8%); 46 (7)	**0.005**
	Yes	4 (1.5%); 16 (0)		6 (2.2%); 22 (26)	
Skin temperature probe	No	183 (67.8%); 43 (9)	**<0.001**	208 (77.0%); 46 (7)	**0.010**
	Yes	87 (32.2%); 39 (11)		62 (23.0%); 44 (8)	
Phototherapy	No	242 (89.6%); 42 (9)	**0.006**	263 (97.4%); 46 (8)	**0.040**
	Yes	28 (10.4%); 36 (12)		7 (2.6%); 41 (5)	
Pulseoximeter sensor	Yes	270 (100%); 41 (10)	-	270 (100%); 46 (8)	-
Carbon-dioxide sensor	No	228 (84.4%); 42 (8)	**< 0.001**	251 (93.0%); 46 (7)	**0.001**
	Yes	42 (15.6%); 35 (11)		19 (7.0%); 41 (9)	
PPS:NICU total score	Overall	41 (10)		46 (8)	**<0.001**

Bold values: statistically significant; IQR: interquartile range.

**Table 3:** Independent variables predicting parental participation in care (n = 270) at T0.

Independent Variable		B	β	95% CI		*p* Value
Birth weight		0.003	0.284	0.001	0.005	**0.002**
Gestational age	Extremely preterm vs. term	−5.588	−0.104	−10.127	−1.050	**0.016**
	Very preterm vs. term	−4.526	−0.160	−6.943	−2.108	**<0.001**
	Late/Moderate preterm vs. term	−0.722	−0.033	−2.605	1.162	0.452
Peripheric venous catheter		1.403	0.075	−0.382	3.188	0.123
Central venous catheter		−0.384	−0.016	−3.150	2.382	0.785
Umbilical venous catheter		−0.851	−0.035	−3.698	1.997	0.557
Continuous infusions		1.739	0.094	−0.350	3.828	0.103
Arterial catheter		−2.864	−0.042	−9.338	3.609	0.385
Ventilatory support	Non-invasive ventilation vs. High-flow nasal cannula	−0.371	−0.008	−4.361	3.619	0.855
	Invasive ventilation vs. High-flow nasal cannula	−3.028	−0.113	−5.541	−0.514	**0.018**
	High-frequency oscillatory ventilation vs. High-flow nasal cannula	−2.251	−0.042	−7.307	2.804	0.382
Gastric tube		0.967	0.051	−0.927	2.861	0.316
Cerebral function monitoring		−2.302	−0.052	−5.857	1.253	0.204
Bladder catheter		−4.849	−0.136	−8.734	−0.963	**0.015**
Stoma		−13.942	−0.206	−19.459	−8.425	**<0.001**
Skin temperature probe		1.331	0.065	−1.217	3.880	0.305
Phototherapy		−3.372	−0.091	−6.741	−0.002	**0.050**
Carbon-dioxide sensor		−0.669	−0.023	−3.813	2.474	0.676
Parents’ gender		−0.364	−0.199	−0.524	−0.205	**<0.001**
Parents’ age		0.037	0.030	−0.061	0.135	0.458
First child		1.361	0.074	−0.190	2.911	0.085
Previous abortions		2.055	0.093	0.259	3.850	**0.025**
Previous deceased children		0.625	0.011	−3.880	5.151	0.782
Type of delivery	Elective cesarean section vs. Natural birth	−2.838	−0.140	−4.660	−1.016	**0.002**
	Emergency cesarean section vs. Natural birth	−3.031	−0.142	−4.937	−1.125	**0.002**
Parents’ ethnicity	African vs. Caucasian	−8.579	−0.234	−11.598	−5.560	**<0.001**
	Hispanic/Latin American vs. Caucasian	2.910	0.060	−1.077	6.897	0.152
Twins		2.112	0.075	−0.631	4.855	0.131
Parents’ employment status	Unemployed vs. Employed	0.836	0.038	−1.086	2.759	0.393
	Student vs. Employed	4.007	0.183	2.101	5.913	**<0.001**
	R^2^	0.263				
	Adjusted R^2^	0.229				

Bold values: statistically significant; R^2^: coefficient of determination; B: unstandardized coefficient; β: standardized coefficient: 95% CI: confidence interval.

## Data Availability

The dataset used in the current study is available in ZENODO and consultable using the identifier 10.5281/zenodo.11071027.

## Supplementary Materials

The following supporting information can be downloaded at: https://www.mdpi.com/article/10.3390/nursrep14020092/s1; Table S1: Italian version of the scale ‘Parental Participation in Care: Neonatal Intensive Care Unit (PPCS: NICU)’.

## Public Involvement Statement

There was no public involvement in any aspect of this research.

## Guidelines and Standards Statement

This study was designed following the statement ‘Strengthening the Reporting of Observational Studies in Epidemiology (STROBE). von Elm, E.; Altman, D.G.; Egger, M.; Pocock, S.J.; Gøtzsche, P.C.; Vandenbroucke, J.P. The Strengthening the Reporting of Observational Studies in Epidemiology (STROBE) Statement: Guidelines for Reporting Observational Studies. Journal of Clinical Epidemiology 2008, 61, 344–349, doi:10.1016/j.jclinepi.2007.11.008.
