# Supplementary material for: Parents’ Participation in Care during Neonatal Intensive Care Unit Stay in COVID-19 Era: An Observational Study
Source: Nurs Rep. 2024 May 13;14(2):1212–23. doi: 10.3390/nursrep14020092 (PMC11130904; doi:10.3390/nursrep14020092)
Supplement: Supplementary file 1 [file nursrep-14-00092-s001.zip › nursrep-2928508-supplementary.pdf]

Italian version of the scale 'Parental Participation in Care: Neonatal Intensive Care Unit (PPCS: NICU)"

N.	ITEM	1 = mai	2 = a volte	3 = sempre
1	il genitore comunica con il personale sanitario			
2	il genitore pone domande riguardo l'assistenza ed il trattamento di suo/a figlio/a			
3	il genitore è disponibile a partecipare ad addestramenti erogati nella Terapia Intensiva Neonatale			
4	il genitore desidera visitare il/la proprio/a figlio/a			
5	il genitore desidera avere contatto fisico con il/la figlio/a (accarezzare, tenere le mani, ecc.)			
6	il genitore usa termini affettivi con suo/a figlio/a			
7	il genitore desidera tenere in braccio il/la figlio/a			
8	il genitore prova a calmare il/la proprio/a figlio/a (cullandolo, cantando, ecc.)			
9	il genitore è attento a posizionare il/la figlio/a in una posizione comoda e consona			
10	il genitore partecipa all' alimentazione del figlio/a			
11a	la mamma è disposta ad allattare al seno			
11b	il papà supporta la mamma affinché il figlio/a riceva latte materno			
12	il genitore partecipa alle cure igieniche del figlio/a			
13	il genitore si accorge di qualsiasi peggioramento nelle condizioni generali del figlio/a			
14	il genitore desidera eseguire il contatto pelle a pelle o desidera partecipare alla kangaroo care con suo figlio/a			
15	il genitore supporta suo figlio/a durante procedure dolorose			
16	il genitore esterna le proprie emozioni e i propri pensieri			
	**PUNTEGGIO TOTALE**			
